# Health-Related Quality of Life in People Living With HIV With Cognitive Symptoms: Assessing Relevant Domains and Associations

**DOI:** 10.1177/23259582231164241

**Published:** 2023-03-22

**Authors:** Kate Alford, Sube Banerjee, Stephanie Daley, Elizabeth Hamlyn, Daniel Trotman, Jaime H. Vera

**Affiliations:** 1 Department of Global Health and Infection, 12190 Brighton and Sussex Medical School, Brighton, UK; 2 Faculty of Health, 6633 University of Plymouth, Plymouth, UK; 3 Centre for Dementia Studies, 12190 Brighton and Sussex Medical School, Brighton, UK; 4 111990 HIV and Sexual Health Service, King's College Hospital NHS Foundation Trust, London, UK; 5 8721 HIV and Sexual Health Service, University Hospitals Sussex NHS Trust, Brighton, UK

**Keywords:** HIV, cognitive impairment, HIV-associated neurocognitive disorder, health-related quality of life, quality of life, patient-centred care

## Abstract

This study aimed to validate and assess a comprehensive set of illness-specific health-related quality of life (HRQL) domains in people living with HIV (PLWH) with cognitive symptoms. One hundred and three HIV patients with cognitive symptoms (*n* = 93 male, 90.3%) were identified from two UK HIV clinics and complete a series of validated scales measuring seven HRQL domains identified as important to HRQL by PLWH with cognitive impairment. These included: physical functioning, cognition, social connectedness, self-concept, HIV stigma, acceptance of and perceived control over cognitive health, and physical and mental health and wellbeing. Exploratory factor analysis confirmed that domain total scores loaded onto one main factor, representing HRQL. Scale cut-off scores revealed a significant proportion of patients scored outside the normal range on single domains (between 26.2% and 79.6%), and many patients on multiple domains (40.8% on 4 or more domains). We found evidence of poor HRQL across domains in the majority of PLWH with cognitive symptoms and identified domains driving these experiences. This provides targets for intervention development and clinical action to maintain or improve HRQL in PLWH with cognitive symptoms or impairment.

## Introduction

People living with HIV (PLWH) experience disproportionately more co-morbidities than age-matched HIV-negative individuals.^1^ These co-morbidities include cognitive impairment which is seen at higher rates and at younger ages in PLWH compared to HIV-negative controls.^1,2^ Conservative estimates suggest between 14% and 28% of PLWH in the UK have a cognitive impairment^3^ and studies find PLWH with cognitive impairment report lower quality of life than PLWH without cognitive impairment and HIV-negative controls.^4^ Cognitive impairment in the context of HIV is often termed HIV-associated neurocognitive disorder (HAND) based on Frascati criteria.^5^ In the last decade, however, there has been increasing recognition that the cognitive impairments seen in PLWH are frequently multifactorial,^6^ and not restricted to brain injury caused directly by HIV and, as such, may not be best described as purely ‘HIV-associated’.^6,7^ Prior to the advent of effective cART, dementia related to HIV disease was commonly seen, but now milder cognitive phenotypes predominate.^7–9^ Importantly, older age appears to increase the risk of cognitive impairment in PLWH, suggesting that the prevalence of cognitive impairment may increase as populations of PLWH age.^10,11^ For many PLWH, the cognitive impairment faced is often static and improvement in cognition is difficult to achieve.^12^ Pharmaceutical interventions which directly target improvement in cognition do not exist, therefore, focusing on broader indicators of wellbeing, such as quality of life, may help to support patients live well with cognitive impairment.

Health-related quality of life (HRQL) is a multidimensional concept that reflects an individual's subjective perception of the impact of a health condition on everyday life and includes domains related to physical health, mental health and social functioning.^13^ The importance of addressing HRQL in PLWH has received increased focus in recent years, as research finds PLWH with virologically controlled HIV infection report substantially lower HRQL compared to the general population.^14–17^ HRQL is directly associated with clinically relevant outcomes in PLWH, including viral suppression and treatment adherence.^18^ As noted above, PLWH with cognitive impairment report poorer HRQL than PLWH without CI, and these individuals face further compounding difficulties.^4,19,20^ The deficits in memory, learning, attention, and executive function experienced have an illness-specific impact on health and function across traditional HRQL domains (i.e., physical, mental, social) along with domains not typically considered in generic or HIV-specific conceptualisations.^19,21–23^ To the best of our knowledge no studies to date have assessed the HRQL of PLWH with cognitive issues along relevant illness-specific domains.

Assessment of HRQL is considered a key measurement of state and outcome in those with chronic diseases, such as HIV or cognitive disorder^24^ and an important factor when evaluating health status in PLWH.^25,26^ Improving HRQL is a recognised goal of treatment. Assessing and targeting illness-specific domains driving HRQL is important for tailored care as it enables healthcare providers to focus on individual needs and illness-specific issues of those experiencing a particular condition during consultation. Systematic assessment of changes in relevant HRQL domains over time can also serve to monitor and evaluate interventions and quantify returns on healthcare investments.^23,27^

In a qualitative study Alford et al^19^ identified important illness-specific domains influencing HRQL in PLWH with objective cognitive impairment (diagnosed in specialist HIV-memory services in two UK hospitals). PLWH reported key issues in domains of: physical function, cognition, social connectedness, HIV stigma, self-concept, acceptance of and control over cognitive health, and physical and mental health and wellbeing.^19^ Using a set of existing instruments selected to represent each HRQL domain identified, this study aimed to further validate the domains influencing HRQL in a larger group of PLWH with cognitive symptoms, explore the associations between domains and to assess the HRQL of this group.

## Methods

### Design and Participants

This questionnaire-based cross-sectional study was approved the [details omitted for double-anonymised peer review] in May 2020. PLWH with access to cART were recruited from two HIV services in [details omitted for double-anonymised peer review] between January and June 2021. Eligible participants were identified based on the European AIDS Clinical Society (EACS) recommended screening tool for the identification of cognitive symptoms (Table 1).^28^ This identifies patients which merit undergoing further investigation and formal neuropsychological testing for potential cognitive impairment. Patients are asked these questions annually as part of their routine care within the HIV service and were identified as having cognitive symptoms if they answered ‘yes’ to one or more of these questions, as recommended by EACS guidance.

**Table 1.** European AIDS Clinical Society Screening Questions for Cognitive Impairment.^28^

Question
1. Do you experience frequent memory loss (e.g., do you forget the occurrence of special events even the more recent ones, appointments, etc.)?
2. Do you feel that you are a lot slower when reasoning, planning activities, or solving problems?
3. Do you have major difficulties paying attention (e.g., to a conversation, book or film)?

### Procedure

Eligible patients were contacted by their HIV service and invited to participate, if interested their contact details were passed to the research team. Participants could decide whether to participate in person or over the telephone and using an electronic link to access and complete the domain scales (Qualtrics^29^). Written or electronic consent was taken before participants completed a demographic questionnaire and the researcher administered Montreal Cognitive Assessment – Blind Version (MoCA-Blind),^30^ this is an adapted version developed and validated for remote administration. This version was used due to the COVID-19 pandemic and allowed those who would prefer to take part remotely to do so.

### Measures

The instruments chosen to represent and ‘operationalise’ the HRQL domains identified^19^ had to fulfil several criteria. This included: i) shown to have acceptable reliability and validity, preferably tested with HIV patients previously; ii) reasonably short in terms of time to complete; and iii) preferable provide (clinically) relevant cut-off points. Details of the instruments used are given below Table 2.

**Table 2.** Scales Employed to Operationalise and Assess HRQL in PLWH With Cognitive Symptoms.

HRQoL domain assessed	Measurement Used	Scale/Scoring
Physical functioning	Lawson and Brody Activities of Daily Living ^31^	Score ranging from 0 (low function, dependent) to 16 (high function, independent).
Cognition	Montreal Cognitive Assessment-Blind (B-MoCA) ^30^	0–22: higher scores indicate higher cognitive functioning. Optimum cut-off of ≤18 for mild CI
Social connectedness	Social Connectedness Scale-Revised (SCS-R) ^32^	Scores range from 20 to 120, with higher scores reflecting a stronger sense of social connectedness.
Mental and physical health and wellbeing	Short-Form-12 Health Survey (SF-12) ^33^	Derives two subscales: physical health and mental health. Scores range from 0 to 100 (with higher scores indicating better health). Population norm of 50.
HIV Stigma	Stigma Scale for Chronic Illness-8^34^	Assesses internalised and enacted stigma. Scores range from 8–40, with higher scores indicating higher stigma.
Self-concept	Rosenburg Self-Esteem Scale^34^	Scores range from 0 to 40, with lower scores indicating worse self-esteem. Scores ≤15 indicate low self-esteem.
Acceptance of and perceived control over cognitive health	Acceptance of Illness Scale (AIS) ^35^	Scores range between 8 and 40, with lower scores indicative of a lack of adjustment to disease, mental discomfort, and overall, less acceptance of the CI
	Brief Illness Perception Scale ^36^	Scores from one dimension: personal control over illness^36^. Range 0–100. Higher scores are representative of less perceived control over illness outcomes.

#### Physical Function

was examined using The Lawson and Brody Instrumental Activities of Daily Living (IADL) Scale.^31^ This measures eight activities of daily living which contributed to the physical function domain. Each activity is scored trichotomously (0 = unable, 1 = needs assistance, 2 = independent) with a summary score ranging from 0 (low function, dependent) to 16 (high function, independent).

#### Cognition

was assessed using the Montreal Cognitive Assessment- Blind (MoCA- Blind).^30^ The adapted MoCA-Blind^30^ has been reported in a number of studies to have good psychometric properties for the screening of cognitive impairment^37,38^ and is shown to be feasible and valid with an optimum cut off of ≤18 (out of a possible 22) for cognitive impairment.^38^

#### Social connectedness

was measured using the Social Connectedness Scale-Revised (SCS-R).^32^ It has excellent psychometric properties as a measure of perception of social inclusion and connection.^39^ Scores range from 20 to 120, with higher scores reflecting a stronger sense of social connectedness.^32^

*HIV stigma* was assessed using the 8-item Stigma Scale for Chronic Illness Scale.^40^ The measure has two subscales assessing internalised and enacted stigma and has been used and validated in PLWH and in people with neurological conditions .^40,41^ Scores range from 8 to 40, with higher scores indicating higher stigma.^34^

*Self- Concept*. was assessed using the Rosenburg Self-Esteem Scale,^34^ a widely used measure of self-esteem. Consisting of 10 items, it has been used and validated extensively for use in PLWH.^42^ Scores range from 0–30, with scores between 15–25 considered normal, and scores below 15 suggestive of low self-esteem.^43^ Self-concept is a multidimensional concept incorporating aspects such as self-efficacy, self-esteem, identity, and future self.^44^ For brevity, we examined one, key area of self-concept, namely self-esteem. This was selected for assessment as self-esteem represents a prominent affective aspect of self-concept: an individual's belief about his or herself,^45^ and thus was considered the most appropriate assessment choice for influencing HRQL.

#### Acceptance of and perceived control over cognitive health

was assessed using two independent scales: the Acceptance of Illness Scale^35^ and the Brief Illness Perception Scale.^36^ The Acceptance of Illness Scale^35^ has been used and validated in PLWH^46^ and in those with neurological conditions.^47^ Scores range between 8 and 40, with lower scores indicative of a lack of adjustment to disease, mental discomfort, and overall less acceptance of the condition.^35^ The Brief Illness Perception Scale^36^ has 8 subdomains capturing 8 dimensions of illness perceptions. We used scores from one dimension: personal control over illness.^36^ It is scored from 0 to 100, with higher scores representative of less perceived control over illness outcomes. The scale performs well psychometrically and has been used in HIV populations ^48^ and in those with cognitive disorder.^49^

#### Mental and physical health and wellbeing

was captured using the two subscales of the Short-Form-12.^33^ This measure has strong validity and reliability in this HIV populations^50^ and assesses health status and HRQL along two subscales: physical health and mental health. These were employed individually to represent the physical and mental health and wellbeing

domain.^51^ Scores range from 0 to 100 (reflecting worse to best health). Scores were analysed on the 0−100 scale, with a cut-off population norm criterion of 50.^33^

*WHOQOL-BREF*^52^. is a well-established instrument measuring generic quality of life widely used in a range of chronic diseases and cancer.^53^ We used the score on the single-item question ‘How would you rate your quality of life?’. Rated on a five-point scale (worse to best). We used the standard recall period of 2 weeks.^52^ Studies have shown that single-item scales, particularly those which ask about concepts whereby the respondents typically have an intuitive understanding, like HRQL, yield good psychometric properties: correlating well with an overall total score or sub-domain score.^54^

### Data Analysis

Data were screened for outliers, normality of distribution, and missing data. Descriptive statistics were performed for the demographic and clinical variables collected. Total *or* sub-scale scores from each HRQL domain scale were computed (based on existing scale specific specifications). Internal reliability was assessed using Cronbach's alpha. The domains identified as comprising HRQL in PLWH with cognitive symptoms were assessed independently of influencing factors such as gender or other social or clinical variables.

Total scores from each domain scale were calculated and exploratory factor analysis (EFA) with parallel analysis^55^ was conducted to assess the validity of the domains identified. Parallel analysis is a Monte Carlo simulation technique that aids researches in determining the number of factor to retain in EFA, extracting factors when the eigenvalue was greater (at the 95^th^ percentile) than that obtained at random.^55^ We hypothesised that scores on the HRQL domain scales would load primarily onto one factor representing overall HRQL. Correlations were performed between HRQL domain scales and across HRQL domain scales, and overall quality of life score as indicated by the single-item measure from the WHOQOL-BREF.^52^ To assess the HRQL of this group in these relevant domains, we examined scores relating to clinical cut-off scores. Where clinical cut-off scores were not available, we used quartiles to divide participants into four groups to determine upper and lower limits allowing insight into the distribution of patients. We examined how many participants scored outside the desired range on each domain scale (i.e., upper or lower quartile (depending on the scale)). To explore the individual and total relevance of the HRQL domains, we performed hierarchical linear regression analyses on the single item from WHOQOL-BREF^52^ first adding cognitive function.^30^ We then added the remaining HRQL domain scales scores to explore the individual associations between HRQL domains scores on overall QoL score.

## Results

### Participants

One hundred and forty-three PLWH with cognitive symptoms were approached to participate, of these 104 PLWH with cognitive symptoms completed the demographic questionnaire and cognitive assessment. One participant failed to complete the HRQL scales online, resulting in a final total of 103 participants. Participants were patients accessing HIV services at [details omitted for double-anonymised peer review] (96 patients, 98.9% and 7 patients, 7.2%, respectively). Ninety-three (90.3%) of participants identified as male; median age was 58.8 years (range 33-88); mean score on the MoCA-Blind was 17.85 (≤18 indication for CI) (Table 3). Those scoring below this threshold (*n* = 52) were referred to HIV-specialist memory clinics, of which 23 received were found to have objective cognitive impairment, 8 were found not to have objective cognitive impairment; and 22 are awaiting assessment.

**Table 3.** Participant Demographics and Clinical Characteristics.

Variable (*n*)	
**Age in years (range)** ^a^	58.8 (32−88)
**Male (%)**	93 (90.3)
**Women (%)**	10 (9.7)
**Race/Ethnicity**	
White – British (%)	66 (64.1)
Black – African (%)	11 (10.7)
White – Other (%)	19 (18.4)
Other (%)	7 (6.8)
**Sexuality**	
MSM (%)	75 (72.8)
Heterosexual (%)	23 (22.3)
Other (%)	5 (4.9)
**Relationship Status**	
Single (%)	55 (53.4)
In a relationship (%)	16 (15.5)
Married/Civil Partnership (%)	32 (31.1)
**Employment**	
Full or part time employed (%)	29 (28.1)
Unemployed (%)	40 (38.8)
Retired (%)	34 (33)
**Average household income per year**	
Less than £20,000 (%)	55 (53.4)
£21,000–30,000 (%)	23 (22.3)
£31,000–£50,000 (%)	14 (13.6)
£51,000–£80,000 (%)	4 (3.9)
More than £80,000 (%)	5 (4.9)
**Education (highest level)**	
Less than 12 years schooling (%)	9 (8.7)
Secondary (left at 16 years of age) (%)	30 (29.1)
College (left at 18 years of age) (%)	31 (30.1)
Degree (Undergraduate/postgraduate degree (%))	33 (32.1)
**Health/HIV clinical variables**	
**MoCA-Blind score (SD)** ^b^	17.85 (3.12)
**Polypharmacy (≥3 non-HIV medications, %)**	67 (65)
**Years with diagnosed HIV** ^a^	19 (2-36)
**Years on ART** ^a^	15 (2-31)
**VL > 40 copies/ml (%)**	5 (5)
**On cART (%)**	103 (100)

MSM, men who have sex with men; MoCA, Montreal Cognitive Assessment; cART, combination antiretroviral therapy; VL, viral load. All values are expressed as *n,* unless otherwise stated.

^a^ Median (range).

^b^ Mean (standard deviation).

### Validation of the HRQL Domain Scales in PLWH With Cognitive Symptoms

We found no outliers across scores on HRQL domains scales and no evidence of non-normality within scale scores. The percentage of missing data totalled 1.69%, which is considered well within acceptable limits. All HRQL scales showed had high internal reliability, all *α* ≥ 0.7,^56^ apart from the MoCA (*α* = 0.62) which was still acceptable.^57^ Used together, the HRQL domain scales also demonstrated good internal consistency (*α* = 0.72).

EFA with parallel analysis indicated a one-factor solution.^55^ The forced one-factor solution explained 44.5% of the variance in scores with some residual correlations remaining, causing model misfit between the following scales: physical health ∼ mental health and self-concept ∼ social connectedness. A confirmatory model with one general factor fitted the data well according to root mean square error of approximation (RMSEA) = 0.007, comparative fit index (CFI) = 0.926, and standardised root mean squared error (SRMR) = 0.064 (*χ*^2^ = 536.54, df = 45, *p* < 0.001). All of the HRQL domains identified displayed factors loadings above the accepted heuristic of ≥0.4^58^ indicating the relevance of each domain to the single factor (HRQL). Contributions ranged from *β* = 0.75 (mental health and control over health outcomes) to *β* = 0.4 (physical health), supporting our conceptualisation of HRQL in PLWH with cognitive impairment.

The single-item overall quality of life score was significantly related to each of the HRQL domain scales in the directions expected. Specifically: higher cognition *(r *= 0.36 [0.16, 0.52]); better physical function (*r *= 0.53 [0.39, 0.65]), higher physical health score (*r *= 0.36 [0.14, 0.57]) and mental health score (*r *= 0.47 [0.27, 0.65]); better self-concept (*r *= 0.47 [0.27, 0.65]); lower perceived HIV stigma (*r *= -0.48 [-0.64, −0.28]); greater acceptance of cognitive impairment (*r *= 0.51 [0.33, 0.6]), and higher perceived control over cognitive health (*r *= -0.61 [-0.72, −0.48) were all significantly correlated with higher reported quality of life. Additionally, we found significant correlations between the majority of HRQL domains. The only domains which did not significantly correlate were cognition and social connectedness, cognition and self-concept, cognition and physical health, and physical health and mental health (all *p *> 0.05) (Table 4).

**Table 4.** Correlations Between HRQL Domain Scales and Overall Quality of Life (WHOQOL-BREF) in People Living With HIV and Cognitive Symptoms.

	1	2	3	4	5	6	7	8	9
1. Quality of life	–	–							
2. Cognition	0.36	–							
3. Physical function	0.53	0.45	–						
4. Social connectedness	0.4	0.07	0.42	–					
5. HIV stigma^a^	−0.48	−0.31	−0.44	−0.32	–				
6. Self-concept	0.47	0.13	0.46	0.74	−0.39	–			
7. Acceptance of CI	0.51	0.4	0.51	0.44	−0.49	0.51	–		
8. Physical health	0.36	0.16	0.3	0.28	−0.23	0.26	0.39	–	
9. Mental health	0.57	0.28	0.46	0.63	−0.51	0.73	0.54	0.07	–
10. Perceived control over cognitive health^a^	−0.54	−0.41	−0.49	−0.36	0.47	−0.32	−0.61	−0.51	−0.47

*p* = <0.05 in bold.

The minus sign (-) indicates that some of the correlations are negative.

^a^ Indicates scale is scored better to worse.

### Health-Related Quality of Life Among People Living With HIV With Cognitive Symptoms

Using cut-off scores or lower or upper quartiles, only 4 (3.9%) participants scored within the desired range on all the HRQL domain scales; 17 (16.5%) participants scored outside the desired range on one scale, 27 (26.2%), 13 (12.6%), 5 (4.9%), 11 (10.7%), 10 (9.7%), 4 (3.9%), scored sub-optimally on 2, 3, 4, 5, 6 scales, respectively. 8 (7.8%) participants scored below desired range on 8 HRQL scales, and 3 (2.9%) scored outside of the desired range on all HRQL scales (Table 5).

**Table 5.** Mean Scores, Standard Deviations, Cut-Off Scores and Number and Percentage of PLWH With Cognitive Symptoms Scoring Sub-Optimally on HRQL Domain Scales.

HRQoL domain (scoring range)	Mean	SD	Cut-off^a^	Number suboptimal	% suboptimal
					
Physical function **(0-16)**	13.47	2.92	Q1:12^+^	34	33
Cognitive functioning **(0-22)**	17.85	3.12	18	52	50.5
Social connectedness **(20-120)**	75.33	19.1	Q1:63^+^	29	28.4
Physical health **(0-100)**	39.66	11.71	50	82	79.6
Mental health **(0-100)**	42.76	13.5	50	70	68
HIV stigma **(8-40)**^a^	18.08	8.44	Q4:22^+^	28	27.7
Self-concept **(0-40)**	23.85	6.33	15	34	33
Acceptance of CI **(8-40)**	31.56	8.85	Q1:25^+^	26	26.5
Perceived control over CI **(0-100)**	36.2	15.59	Q4:45^+^	27	26.2

SD, standard deviation.

^a^ Higher scores indicate worse outcome.

^+^Based on literature, if not available based on lower (Q1) or upper (Q4) quartile.

Overall quality of life (single WHOQOL-BREF item) was significantly predicted through hierarchical linear regression by cognitive function (Step 1) (see Table 5 for full details of each regression model) *R*^2^*^ ^*= 0.14, *F*(1, 91) = 15.15, *p *< 0.001, Cohens *f*^2 ^= 0.16. The addition of the remaining HRQL domain scale scores (Step 2) led to a statistically significant increase in *R^2^* of 0.56, *F* (8,83) = 11.76, *p *< 0.001, Cohen's *f*^2 ^= 0.79. Physical function, physical health, mental health, and control over health outcomes were the strongest predictors of HRQL in the final model (all *p *< 0.05) (Table 6).

**Table 6.** Linear Model of Prediction of QoL from HRQL Domains Scales in PLWH With Cognitive Symptoms.

	*b*	***SE B***	***β***	***p***
**Step 1**				
**Constant**	2.31 [1.63, 2.99]	0.34		
**Cognition**	0.85 [0.41, 1.28]	0.22	0.38	***p* < 0.001**
**Step 2**				* *
**Constant**	0.64 [0.86, 2.14]	0.75		* *
**Cognition**	0.09 [-0.18, 0.57]	0.18	0.09	*p* = 0.31
**Physical function**	0.09 [0.02, 0.17]	0.04	0.24	***p *= 0.02**
**Social connectedness**	-0.01 [-0.02, 0.01]	0.01	-0.11	*p* = 0.35
**Physical health**	0.03 [0.01, 0.05]	0.01	0.27	***p* < 0.01**
**Mental health**	0.03 [0.01, 0.05]	0.01	0.36	***p* < 0.01**
**HIV stigma**	-0.01 [-0.04, 0.01]	0.01	-0.09	*p* = 0.31
**Self-concept**	0.02 [-0.03, 0.06]	0.02	0.09	*p *= 0.47
**Acceptance of CI**	0.01 [-0.01, 0.04]	0.01	0.11	*p *= 0.32
**Control over health outcomes**	0.06 [0.00, 0.11]	0.03	0.17	***p *< 0.01**

*R*^2 ^=* *0.14 for Step 1; Δ*R*^2 ^= 0.41 for step 2 (*ps* < 0.001); *p* < 0.05 in bold.

## Discussion

In this study, we examined a comprehensive set of HRQL scales, selected to represent the domains (physical function, cognition, social connectedness, physical and mental health and wellbeing, self-concept, HIV stigma, and acceptance of cognitive impairment and control over cognitive health outcomes) identified as comprising HRQL in PLWH with cognitive impairment.^19^ The findings confirmed the relevance of the domains in a larger group of PLWH with cognitive symptoms as scores loaded primarily onto one common factor. This indicated that there is one underlying construct driving scores and that each domain fits onto this single theoretical construct, which we identified as HRQL based on previous qualitative work.^19^ This is further supported by significant correlations between all the HRQL domain scores on the single-item overall quality of life score and significant correlations between most domains (*r* 0.73 to −0.74, *p *< 0.05), suggesting attention to any of the domains could inform interventions to improve HRQL. Exploring cut-off scores revealed a significant proportion of PLWH with cognitive symptoms scored outside the desired range on single HRQL domains (between 26.2% and 79.6%). Moreover, the vast majority (96.1%) of PLWH with cognitive symptoms scored outside the desired range on at least one or more of the HRQL domains, and many scored sub-optimally on four or more domains (40.8%). This shows the scope for improvement in HRQL in PLWH with cognitive symptoms.

Regression analysis found cognitive function significantly predicted quality of life score (Step 1 on regression model). Adding in the remaining HRQL domain measures significantly improved model fit and increased the percentage of explained variance to 56% and underscores the relevance of the domains in this population. Our findings are in line with studies that have used similar methods to quantitatively operationalise domains considered important to HRQL such as the study of a population of Dutch PLWH which found that 51.2% of variance was explained in their model.^59^ The authors of this study argued that the regression model produced indicates the predictive efficacy of their scales and suggested their scale battery be employed within Dutch HIV clinics to measure HRQL.^59^

We found that domains of physical function, physical health, mental health, and control over health outcomes were significantly associated with the single-item quality of life score in the final regression model (all *p *< 0.05). Interestingly, social connectedness, stigma, self-concept and acceptance of CI were not statistically significant predictors of quality of life. Literature shows that these domains are strongly associated with HRQL in PLWH^60,61^ and with those experiencing cognitive disorders,^27,62^ which was further evidenced in the correlational analyses presented whereby each domain showed a small-medium effect (*r* between 0.4 and 0.51) on the quality of life score. A further regression analysis indicated when controlling for the other domains the inclusion of these domain scales increased the percentage of explained variance by only 3% (Δ*R*^2 ^= 0.03). Therefore, from the perspective of the regression analysis, conceptually these domains add little unique variance.

This is the first study to measure illness-specific domains of HRQL among PLWH with cognitive symptoms and compliments work conducted by Vance et al.^63^ Corroborating qualitative work^19^ we have quantitatively demonstrated the relevance of domains important to HRQL in PLWH with cognitive symptoms through factor analysis, regression, and correlational analyses. Given no interventions exist to improve cognition for the majority of PLWH experiencing cognitive symptoms, focusing on improving broader indicators of wellbeing is important. The findings from this study provide information for clinicians to consider when assessing HRQL in patients reporting cognitive symptoms, targets for intervention development to improve quality of life in this group, and signposting information regarding the type of community services which may benefit PLWH with cognitive symptoms. A second strength is that by using clinical-cuts off and the upper and lower quartiles of scales, we have demonstrated the particularly poor HRQL in this group in the absence of an HIV-negative control group, and give weight to the importance of developing interventions to improve HRQL. A third strength of this study was the high response rate from eligible patients, decreasing the potential for response bias.

An important limitation of the study is the use of the EACS screening questions to identify the sample. We acknowledge that these questions only indicate those with cognitive symptoms who may benefit from further investigation and yield poor sensitivity and moderate specificity in the detection of cognitive impairment.^64^ Caution should be taken therefore if extrapolating the findings to PLWH with clinically significant cognitive impairment. Despite this, given the value of good HRQL for all PLWH, identifying and assessing HRQL in those that at a minimum experience cognitive symptoms is important for tailored care and overall patient wellbeing. A second limitation of the study was the homogeneity of the sample. Our sample was largely comprised of men who have sex with men (MSM), with few female participants. These distributions reflect the patient cohorts attending services in [details omitted for double-anonymised peer review] (in particular) but do not reflect the distribution of PLWH with cognitive impairment across the wider UK. This limitation to generalisability should be carefully considered when applying findings. It is however notable that PLWH who are MSM tend to report higher HRQL than other PLWH groups.^65^ As such, HRQL in a sample more representative of the UK HIV population might find HRQL to be worse than that reported in this study. Further research is needed in more representative populations. A further limitation is that we did not explore the effects of age, gender, ethnicity, HIV history, severity of cognitive symptoms, or concurrent co-morbidities because of sample size restrictions. The influence of these factors requires examination in the future. Additionally, future research could examine how impairment in different cognitive domains are related to different indicators of HRQL in PLWH with cognitive impairment.

## Conclusion

HRQL is impaired for the majority of PLWH with cognitive symptoms. It could be improved by attending to the domains we have identified to be driving these experiences. The domains were strongly associated with one another, therefore actions to address any could inform interventions to improve HRQL. These data provide targets for intervention development and clinical action to maintain or improve HRQL in PLWH with cognitive symptoms and impairment.
